# Bis[1,2-bis­(dimethyl­phosphino)­ethane]­dichloridonitro­syltungsten(0) chloride

**DOI:** 10.1107/S1600536807067128

**Published:** 2007-12-22

**Authors:** Nataša Avramović, Olivier Blacque, Helmut W. Schmalle, Heinz Berke

**Affiliations:** a Anorganisch-Chemisches Institut der Universität Zürich, Winterthurerstrasse 190, CH-8057 Zürich, Switzerland

## Abstract

In the crystal structure of the title compound, [WCl_2_(NO)(C_6_H_16_P_2_)_2_]Cl, the seven-coordinated tungsten(II) center displays a distorted penta­gonal–bipyramidal geometry with *trans* nitrosyl and chloride ligands. The NO and Cl ligands are disordered over two positions; the site occupancy factors are 0.6 and 0.4.

## Related literature

For related *trans*-chloridonitrosyl-tungsten complexes, see: Chen *et al.* (2007 ▶). For related *trans*-chloridonitrosyl-bis­(1,2-bis­(dimethyl­phosphino)ethane)molybdenum complexes, see: Liang *et al.* (2003 ▶, 2006 ▶). For related literature, see: Bencze & Kohàn (1982 ▶); Carmona *et al.* (1989 ▶); Hunter & Legzdins (1984 ▶).
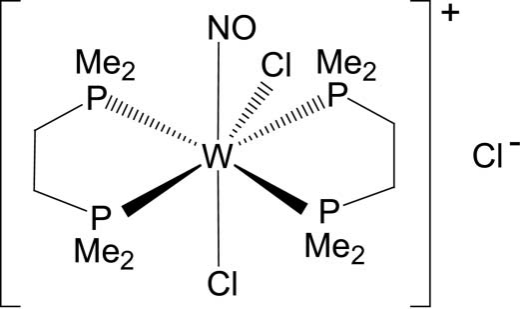


## Experimental

### 

#### Crystal data

[WCl_2_(NO)(C_6_H_16_P_2_)_2_]Cl
*M* *_r_* = 620.46
Monoclinic, 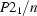

*a* = 8.0929 (7) Å
*b* = 26.118 (2) Å
*c* = 10.5703 (10) Å
β = 94.190 (10)°
*V* = 2228.3 (3) Å^3^
*Z* = 4
Mo *K*α radiation
μ = 5.83 mm^−1^
*T* = 183 (2) K
0.20 × 0.15 × 0.07 mm

#### Data collection

Stoe IPDS diffractometer
Absorption correction: numerical (Coppens *et al.*, 1965 ▶) *T*_min_ = 0.409, *T*_max_ = 0.723
7761 measured reflections
3937 independent reflections
3353 reflections with *I* > 2σ(*I*)
*R*_int_ = 0.035

#### Refinement

*R*[*F* ^2^ > 2σ(*F* ^2^)] = 0.025
*wR*(*F* ^2^) = 0.060
*S* = 0.97
3937 reflections
217 parameters
H-atom parameters constrained
Δρ_max_ = 0.83 e Å^−3^
Δρ_min_ = −1.28 e Å^−3^

### 

Data collection: *IPDS Software* (Stoe & Cie, 1999 ▶); cell refinement: *IPDS Software*; data reduction: *X-RED* in *IPDS Software*; program(s) used to solve structure: *SHELXS97* (Sheldrick, 1997 ▶); program(s) used to refine structure: *SHELXL97* (Sheldrick, 1997 ▶); molecular graphics: *PLATON* (Spek, 2003 ▶); software used to prepare material for publication: *SHELXL97*.

## Supplementary Material

Crystal structure: contains datablocks global, I. DOI: 10.1107/S1600536807067128/su2037sup1.cif

Structure factors: contains datablocks I. DOI: 10.1107/S1600536807067128/su2037Isup2.hkl

Additional supplementary materials:  crystallographic information; 3D view; checkCIF report

## Figures and Tables

**Table:** 

W1—P1	2.5665 (11)
W1—P2	2.5745 (11)
W1—P3	2.5583 (11)
W1—P4	2.5788 (11)
W1—Cl1	2.4935 (11)
W1—Cl21	2.445 (3)
W1—Cl22	2.431 (5)
W1—N11	1.840 (11)
W1—N12	1.845 (13)

**Table:** 

P1—W1—P2	73.07 (4)
P1—W1—P3	76.49 (3)
Cl1—W1—P2	68.84 (4)
Cl1—W1—P4	68.56 (4)
P3—W1—P4	73.37 (4)

**Fig. 1.**
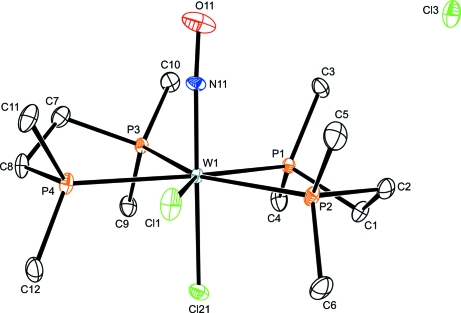
View of the molecular structure of (I) with the atom-labeling scheme (displacement ellipsoids are drawn at the 30% probability level). The disordered atoms N12, O12 and Cl22, and all hydrogen atoms have been omitted for clarity.

## supplementary crystallographic information

### Comment

The title compound [W(Cl)_2_(NO)(dmpe)_2_](Cl) (I) was obtained by the reaction of [W(Cl)_3_(NO)(NCCH_3_)_2_] with 2.5 equivalents of dmpe at room temperature in tetrahydrofurane. The tungsten center has transformed into a seven coordination environment and exhibits a distorted pentagonal bipyramidal geometry, where the four phosphorus atoms and one chloride form the pentagon, and the *trans* nitrosyl and chloride ligands are at the apexes (Figure 1). This geometry is clearly different to that observed for the related compound Mo(Cl)_3_(NO)(PMe_3_)_3_, for which the coordination polyhedron is described as a capped-octahedron (Carmona *et al.*, 1989). The five equatorial atoms, P1, P2, P3, P4, and Cl1 are in an approximately planar array and the corresponding equatorial angles are in the range 68.5 - 76.5°. The two Cl—W—P bond angles of 68.84 (4) and 68.56 (4)° are smaller than the theoretical average angle of 72°, while all three P—W—P angles are larger (73.07 (4) - 76.49 (3)°). The nitrosyl group is located *trans* to one chloride ligand and they are positionally disordered in a ratio 0.6:0.4 (Chen *et al.*, 2007). One chloride ion acts as a counterion and is not coordinated, resulting in a tungsten center in the oxidation state +2.

### Experimental

[W(Cl)_2_(NO)(dmpe)_2_]Cl was prepared from complex [W(Cl)_3_(NO)(CH_3_CN)_2_], which is easily synthesized by the reaction of W(Cl)_6_ with NO gas in dichloromethane in the presence of acetonitrile at room temperature (Bencze & Kohàn, 1982; Hunter & Legzdins, 1984). 5.00 g (12.6 mmol) of WCl_6_ and 1.32 ml (25.2 mmol) of acetonitrile were dissolved in 180 ml of dichloromethane in a 500 ml three-necked flask. Nitric oxide was passed through the solution, which was stirred at room temperature until the dark purple colour of the solution turned to the light green precipitate after *ca* 1 h. The volume of the final mixture was reduced to 50 ml *in vacuo* and the mixture was then cooled to 0°C for 15 min. The precipitate was isolated by filtration and the collected solid was washed with cold dichloromethane (2 *x* 10 ml at 0°C) and then with hexane (4 *x* 20 ml) at room temperature. Final drying of the solid under vacuum for 18 h afforded the yellow-green [W(Cl)_3_(NO)(CH_3_CN)_2_] compound. 0.356 g (0.88 mmol) of [W(Cl)_3_(NO)(NCCH_3_)_2_] was dissolved in 20 ml of tetrahydrofurane in a Young tap Schlenk and the dmpe ligand (0.38 ml, 2.20 mmol) was syringed into the solution. After 24 h of stirring at room temperature, the solution was filtered off and the solvent was removed under vacuum. The resulting precipitate was extracted with dichloromethane and crystallized in dichloromethane at room temperature to give yellow crystals of compound (I).

Yield: 0.491 g (90%).

IR (cm^-1^, CH_2_Cl_2_): 1608 (NO).

^1^H NMR (200.0 MHz, CD_2_Cl_2_, 25°C): d 2.09 (m, 8H, P(C*H*_2_)_2_P), 2.01 (m, 12H, PC*H*_3_) and 1.86 (m, 12H, PC*H*_3_).

^31^P{^1^H} NMR (80.9 MHz, CD_2_Cl_2_, 25°C): d 43.8 (m, *P*(CH_2_)_2_P) and 21.9 (m, P(CH_2_)_2_*P*), ^2^J_PN_ = 27.6 Hz; ^1^J_PW_ = 200 Hz.

^13^C{^1^H} NMR (50.3 MHz, CD_2_Cl_2_, 25°C): d 33.4 (m, P(*C*H_2_)_2_P), 24.3 (m, P(*C*H_2_)_2_P), 13.2 (m, P*C*H_3_) and 11.5 (m, P*C*H_3_).

Anal. Calcd for C_12_H_32_Cl_3_P_4_NOW: C, 23.22; H, 5.16; N, 2.26. Found: C, 23.26; H, 5.28; N, 2.37.

### Refinement

The H atom were included in calculated positions and treated as riding atoms with C—H distances = 0.98 - 0.99Å and *U*_iso_(H) = 1.2*U*_eq_(C) for CH_2_ and 1.5*U*_eq_(C) for the CH_3_ groups. A positional disorder was refined for the *trans* NO and Cl ligands with occupancy factors of 0.6:0.4.

### Crystal data

**Table:**

[WCl_2_(NO)(C_6_H_16_P_2_)_2_]Cl	*F*_000_ = 1216
*M**_r_* = 620.46	*D*_x_ = 1.850 Mg m^−^^3^
Monoclinic, *P*2_1_/*n*	Mo *K*α radiation λ = 0.71073 Å
Hall symbol: -P 2yn	Cell parameters from 8000 reflections
*a* = 8.0929 (7) Å	θ = 2.4–28.0º
*b* = 26.118 (2) Å	µ = 5.83 mm^−^^1^
*c* = 10.5703 (10) Å	*T* = 183 (2) K
β = 94.190 (10)º	Plate, yellow
*V* = 2228.3 (3) Å^3^	0.20 × 0.15 × 0.07 mm
*Z* = 4	

### Data collection

**Table:**

Stoe IPDS diffractometer	3937 independent reflections
Radiation source: fine-focus sealed tube	3353 reflections with *I* > 2σ(*I*)
Monochromator: graphite	*R*_int_ = 0.035
*T* = 183(2) K	θ_max_ = 25.0º
φ oscillation scan	θ_min_ = 2.5º
Absorption correction: numerical(Coppens *et al.*, 1965)	*h* = −9→9
*T*_min_ = 0.410, *T*_max_ = 0.723	*k* = −30→30
7761 measured reflections	*l* = 0→12

### Refinement

**Table:**

Refinement on *F*^2^	Secondary atom site location: difference Fourier map
Least-squares matrix: full	Hydrogen site location: inferred from neighbouring sites
*R*[*F*^2^ > 2σ(*F*^2^)] = 0.025	H-atom parameters constrained
*wR*(*F*^2^) = 0.060	*w* = 1/[σ^2^(*F*_o_^2^) + (0.0359*P*)^2^] where *P* = (*F*_o_^2^ + 2*F*_c_^2^)/3
*S* = 0.98	(Δ/σ)_max_ = 0.001
3937 reflections	Δρ_max_ = 0.83 e Å^−^^3^
217 parameters	Δρ_min_ = −1.28 e Å^−^^3^
Primary atom site location: structure-invariant direct methods	Extinction correction: none

### Special details

**Table:**

Geometry. All e.s.d.'s (except the e.s.d. in the dihedral angle between two l.s. planes) are estimated using the full covariance matrix. The cell e.s.d.'s are taken into account individually in the estimation of e.s.d.'s in distances, angles and torsion angles; correlations between e.s.d.'s in cell parameters are only used when they are defined by crystal symmetry. An approximate (isotropic) treatment of cell e.s.d.'s is used for estimating e.s.d.'s involving l.s. planes.
Refinement. Refinement of *F*^2^ against ALL reflections. The weighted *R*-factor *wR* and goodness of fit *S* are based on *F*^2^, conventional *R*-factors *R* are based on *F*, with *F* set to zero for negative *F*^2^. The threshold expression of *F*^2^ > σ(*F*^2^) is used only for calculating *R*-factors(gt) *etc*. and is not relevant to the choice of reflections for refinement. *R*-factors based on *F*^2^ are statistically about twice as large as those based on *F*, and *R*- factors based on ALL data will be even larger.

### Fractional atomic coordinates and isotropic or equivalent isotropic displacement parameters (Å^2^)

**Table:**

	*x*	*y*	*z*	*U*_iso_*/*U*_eq_	Occ. (<1)
W1	0.14034 (2)	0.138353 (6)	0.190758 (14)	0.01228 (7)	
P1	0.28028 (14)	0.05181 (4)	0.24110 (10)	0.0148 (2)	
P2	0.08089 (15)	0.11528 (4)	0.41984 (10)	0.0187 (2)	
P3	0.28985 (14)	0.11874 (4)	−0.00872 (10)	0.0153 (2)	
P4	0.05122 (15)	0.20805 (4)	0.02694 (10)	0.0201 (2)	
Cl1	−0.03756 (17)	0.20441 (5)	0.28354 (11)	0.0320 (3)	
C1	0.1747 (6)	0.01929 (18)	0.3647 (4)	0.0263 (11)	
H1A	0.0601	0.0104	0.3331	0.032*	
H1B	0.2337	−0.0127	0.3898	0.032*	
C2	0.1720 (6)	0.05517 (19)	0.4784 (4)	0.0258 (11)	
H2A	0.2859	0.0610	0.5164	0.031*	
H2B	0.1050	0.0402	0.5438	0.031*	
C3	0.4913 (6)	0.0544 (2)	0.3091 (4)	0.0266 (11)	
H3A	0.5357	0.0196	0.3182	0.040*	
H3B	0.4941	0.0708	0.3926	0.040*	
H3C	0.5588	0.0742	0.2534	0.040*	
C4	0.2717 (7)	0.00030 (18)	0.1264 (5)	0.0311 (12)	
H4A	0.3430	0.0086	0.0581	0.047*	
H4B	0.1574	−0.0041	0.0908	0.047*	
H4C	0.3102	−0.0315	0.1681	0.047*	
C5	0.1471 (7)	0.1608 (2)	0.5415 (5)	0.0357 (13)	
H5A	0.0950	0.1940	0.5221	0.054*	
H5B	0.2679	0.1644	0.5447	0.054*	
H5C	0.1144	0.1486	0.6237	0.054*	
C6	−0.1376 (6)	0.1041 (2)	0.4354 (5)	0.0323 (12)	
H6A	−0.1580	0.1016	0.5254	0.048*	
H6B	−0.1709	0.0720	0.3924	0.048*	
H6C	−0.2021	0.1325	0.3967	0.048*	
C7	0.3072 (6)	0.17713 (19)	−0.1023 (4)	0.0273 (11)	
H7A	0.3815	0.2019	−0.0554	0.033*	
H7B	0.3546	0.1690	−0.1837	0.033*	
C8	0.1377 (6)	0.19998 (19)	−0.1269 (4)	0.0273 (11)	
H8A	0.0664	0.1770	−0.1818	0.033*	
H8B	0.1450	0.2335	−0.1700	0.033*	
C9	0.1887 (6)	0.07605 (19)	−0.1249 (4)	0.0286 (11)	
H9A	0.2509	0.0755	−0.2011	0.043*	
H9B	0.0757	0.0881	−0.1473	0.043*	
H9C	0.1848	0.0415	−0.0894	0.043*	
C10	0.5051 (6)	0.1013 (2)	0.0067 (5)	0.0287 (11)	
H10A	0.5169	0.0670	0.0435	0.043*	
H10B	0.5664	0.1259	0.0621	0.043*	
H10C	0.5495	0.1016	−0.0771	0.043*	
C11	0.1231 (7)	0.27186 (18)	0.0718 (5)	0.0337 (12)	
H11A	0.0917	0.2959	0.0030	0.051*	
H11B	0.2440	0.2715	0.0876	0.051*	
H11C	0.0725	0.2826	0.1490	0.051*	
C12	−0.1699 (6)	0.2108 (2)	−0.0142 (5)	0.0316 (12)	
H12A	−0.2282	0.2161	0.0627	0.047*	
H12B	−0.2066	0.1785	−0.0545	0.047*	
H12C	−0.1944	0.2392	−0.0732	0.047*	
Cl3	0.54253 (16)	0.09564 (5)	0.66427 (11)	0.0299 (3)	
Cl21	−0.0944 (3)	0.08497 (11)	0.1146 (2)	0.0243 (8)	0.597 (5)
N11	0.3280 (12)	0.1748 (4)	0.2417 (8)	0.020 (2)	0.597 (5)
O11	0.4639 (12)	0.1916 (4)	0.2679 (8)	0.051 (3)	0.597 (5)
Cl22	0.3926 (7)	0.18470 (18)	0.2565 (4)	0.0243 (8)	0.403 (5)
N12	−0.0356 (16)	0.0967 (5)	0.1383 (12)	0.020 (2)	0.403 (5)
O12	−0.1463 (16)	0.0674 (6)	0.1024 (13)	0.051 (3)	0.403 (5)

### Atomic displacement parameters (Å^2^)

**Table:**

	*U*^11^	*U*^22^	*U*^33^	*U*^12^	*U*^13^	*U*^23^
W1	0.01609 (10)	0.01170 (10)	0.00949 (9)	0.00126 (7)	0.00396 (6)	−0.00089 (6)
P1	0.0185 (6)	0.0143 (5)	0.0119 (5)	0.0007 (4)	0.0024 (4)	0.0001 (4)
P2	0.0244 (6)	0.0205 (6)	0.0122 (5)	−0.0016 (5)	0.0078 (4)	−0.0001 (4)
P3	0.0178 (6)	0.0186 (6)	0.0099 (5)	0.0015 (4)	0.0046 (4)	0.0001 (4)
P4	0.0278 (7)	0.0147 (6)	0.0184 (6)	0.0052 (5)	0.0066 (5)	0.0039 (4)
Cl1	0.0477 (8)	0.0254 (6)	0.0252 (6)	0.0177 (6)	0.0176 (5)	0.0007 (5)
C1	0.033 (3)	0.019 (2)	0.027 (3)	−0.002 (2)	0.008 (2)	0.005 (2)
C2	0.029 (3)	0.032 (3)	0.017 (2)	−0.003 (2)	0.0082 (19)	0.0006 (19)
C3	0.021 (2)	0.037 (3)	0.022 (2)	0.007 (2)	−0.0004 (19)	0.004 (2)
C4	0.051 (3)	0.017 (2)	0.025 (3)	0.006 (2)	−0.001 (2)	−0.0013 (19)
C5	0.054 (4)	0.032 (3)	0.021 (2)	−0.004 (3)	0.003 (2)	−0.006 (2)
C6	0.027 (3)	0.037 (3)	0.035 (3)	0.001 (2)	0.016 (2)	0.006 (2)
C7	0.035 (3)	0.027 (3)	0.022 (2)	−0.002 (2)	0.011 (2)	0.000 (2)
C8	0.041 (3)	0.024 (3)	0.017 (2)	0.006 (2)	0.004 (2)	0.0076 (19)
C9	0.038 (3)	0.030 (3)	0.017 (2)	0.001 (2)	0.001 (2)	−0.001 (2)
C10	0.023 (2)	0.040 (3)	0.025 (2)	0.005 (2)	0.0107 (19)	0.002 (2)
C11	0.053 (3)	0.014 (2)	0.035 (3)	−0.001 (2)	0.009 (2)	0.003 (2)
C12	0.031 (3)	0.031 (3)	0.033 (3)	0.011 (2)	0.004 (2)	0.012 (2)
Cl3	0.0378 (7)	0.0303 (7)	0.0229 (6)	0.0086 (5)	0.0107 (5)	0.0059 (5)
Cl21	0.016 (2)	0.0332 (14)	0.0231 (12)	0.0016 (14)	−0.0007 (12)	−0.0017 (10)
N11	0.010 (5)	0.034 (4)	0.017 (4)	−0.002 (4)	0.004 (3)	−0.002 (3)
O11	0.027 (5)	0.073 (5)	0.052 (4)	−0.004 (4)	−0.006 (4)	−0.018 (4)
Cl22	0.016 (2)	0.0332 (14)	0.0231 (12)	0.0016 (14)	−0.0007 (12)	−0.0017 (10)
N12	0.010 (5)	0.034 (4)	0.017 (4)	−0.002 (4)	0.004 (3)	−0.002 (3)
O12	0.027 (5)	0.073 (5)	0.052 (4)	−0.004 (4)	−0.006 (4)	−0.018 (4)

### Geometric parameters (Å, °)

**Table:**

W1—P1	2.5665 (11)	C3—H3B	0.9800
W1—P2	2.5745 (11)	C3—H3C	0.9800
W1—P3	2.5583 (11)	C4—H4A	0.9800
W1—P4	2.5788 (11)	C4—H4B	0.9800
W1—Cl1	2.4935 (11)	C4—H4C	0.9800
W1—Cl21	2.445 (3)	C5—H5A	0.9800
W1—Cl22	2.431 (5)	C5—H5B	0.9800
W1—N11	1.840 (11)	C5—H5C	0.9800
W1—N12	1.845 (13)	C6—H6A	0.9800
N11—O11	1.198 (15)	C6—H6B	0.9800
N12—O12	1.218 (17)	C6—H6C	0.9800
P1—C3	1.805 (5)	C7—C8	1.502 (7)
P1—C4	1.809 (5)	C7—H7A	0.9900
P1—C1	1.823 (5)	C7—H7B	0.9900
P2—C5	1.803 (5)	C8—H8A	0.9900
P2—C6	1.812 (5)	C8—H8B	0.9900
P2—C2	1.823 (5)	C9—H9A	0.9800
P3—C10	1.796 (5)	C9—H9B	0.9800
P3—C9	1.810 (5)	C9—H9C	0.9800
P3—C7	1.829 (5)	C10—H10A	0.9800
P4—C12	1.812 (5)	C10—H10B	0.9800
P4—C11	1.817 (5)	C10—H10C	0.9800
P4—C8	1.829 (5)	C11—H11A	0.9800
C1—C2	1.525 (7)	C11—H11B	0.9800
C1—H1A	0.9900	C11—H11C	0.9800
C1—H1B	0.9900	C12—H12A	0.9800
C2—H2A	0.9900	C12—H12B	0.9800
C2—H2B	0.9900	C12—H12C	0.9800
C3—H3A	0.9800		
			
N11—W1—N12	174.8 (4)	P2—C2—H2A	110.3
N11—W1—Cl21	175.3 (3)	C1—C2—H2B	110.3
N12—W1—Cl22	173.3 (5)	P2—C2—H2B	110.3
Cl22—W1—Cl21	173.87 (10)	H2A—C2—H2B	108.6
N11—W1—Cl1	90.8 (3)	P1—C3—H3A	109.5
N12—W1—Cl1	94.1 (4)	P1—C3—H3B	109.5
Cl21—W1—Cl1	93.84 (7)	H3A—C3—H3B	109.5
Cl22—W1—Cl1	92.29 (11)	P1—C3—H3C	109.5
N11—W1—P3	85.1 (2)	H3A—C3—H3C	109.5
N12—W1—P3	92.0 (4)	H3B—C3—H3C	109.5
Cl22—W1—P3	84.12 (11)	P1—C4—H4A	109.5
Cl21—W1—P3	91.00 (6)	P1—C4—H4B	109.5
Cl1—W1—P3	141.64 (4)	H4A—C4—H4B	109.5
N11—W1—P1	93.0 (3)	P1—C4—H4C	109.5
N12—W1—P1	82.1 (4)	H4A—C4—H4C	109.5
Cl22—W1—P1	91.76 (11)	H4B—C4—H4C	109.5
Cl21—W1—P1	83.48 (6)	P2—C5—H5A	109.5
Cl1—W1—P1	141.86 (4)	P2—C5—H5B	109.5
P1—W1—P2	73.07 (4)	H5A—C5—H5B	109.5
P1—W1—P3	76.49 (3)	P2—C5—H5C	109.5
N11—W1—P2	93.0 (3)	H5A—C5—H5C	109.5
N12—W1—P2	87.2 (4)	H5B—C5—H5C	109.5
Cl22—W1—P2	93.34 (11)	P2—C6—H6A	109.5
Cl21—W1—P2	88.97 (6)	P2—C6—H6B	109.5
Cl1—W1—P2	68.84 (4)	H6A—C6—H6B	109.5
P3—W1—P2	149.37 (4)	P2—C6—H6C	109.5
N11—W1—P4	90.9 (3)	H6A—C6—H6C	109.5
N12—W1—P4	92.5 (4)	H6B—C6—H6C	109.5
Cl22—W1—P4	91.62 (11)	C8—C7—P3	108.6 (3)
Cl21—W1—P4	90.52 (7)	C8—C7—H7A	110.0
Cl1—W1—P4	68.56 (4)	P3—C7—H7A	110.0
P3—W1—P4	73.37 (4)	C8—C7—H7B	110.0
P1—W1—P4	149.15 (3)	P3—C7—H7B	110.0
P2—W1—P4	137.26 (4)	H7A—C7—H7B	108.3
C3—P1—C4	106.5 (2)	C7—C8—P4	107.2 (3)
C3—P1—C1	102.2 (2)	C7—C8—H8A	110.3
C4—P1—C1	97.9 (2)	P4—C8—H8A	110.3
C3—P1—W1	116.09 (17)	C7—C8—H8B	110.3
C4—P1—W1	121.42 (16)	P4—C8—H8B	110.3
C1—P1—W1	109.64 (16)	H8A—C8—H8B	108.5
C5—P2—C6	106.4 (3)	P3—C9—H9A	109.5
C5—P2—C2	103.5 (2)	P3—C9—H9B	109.5
C6—P2—C2	101.7 (2)	H9A—C9—H9B	109.5
C5—P2—W1	116.77 (18)	P3—C9—H9C	109.5
C6—P2—W1	111.95 (17)	H9A—C9—H9C	109.5
C2—P2—W1	114.95 (15)	H9B—C9—H9C	109.5
C10—P3—C9	107.0 (2)	P3—C10—H10A	109.5
C10—P3—C7	98.5 (2)	P3—C10—H10B	109.5
C9—P3—C7	101.3 (2)	H10A—C10—H10B	109.5
C10—P3—W1	119.33 (16)	P3—C10—H10C	109.5
C9—P3—W1	117.72 (17)	H10A—C10—H10C	109.5
C7—P3—W1	109.79 (16)	H10B—C10—H10C	109.5
C12—P4—C11	108.6 (3)	P4—C11—H11A	109.5
C12—P4—C8	103.1 (2)	P4—C11—H11B	109.5
C11—P4—C8	101.6 (2)	H11A—C11—H11B	109.5
C12—P4—W1	114.33 (17)	P4—C11—H11C	109.5
C11—P4—W1	113.81 (17)	H11A—C11—H11C	109.5
C8—P4—W1	114.16 (15)	H11B—C11—H11C	109.5
C2—C1—P1	108.2 (3)	P4—C12—H12A	109.5
C2—C1—H1A	110.1	P4—C12—H12B	109.5
P1—C1—H1A	110.1	H12A—C12—H12B	109.5
C2—C1—H1B	110.1	P4—C12—H12C	109.5
P1—C1—H1B	110.1	H12A—C12—H12C	109.5
H1A—C1—H1B	108.4	H12B—C12—H12C	109.5
C1—C2—P2	106.9 (3)	O11—N11—W1	169.1 (8)
C1—C2—H2A	110.3	O12—N12—W1	176.9 (15)
			
N11—W1—P1—C3	2.3 (3)	Cl22—W1—P3—C9	175.1 (2)
N12—W1—P1—C3	−179.4 (4)	Cl21—W1—P3—C9	−1.2 (2)
Cl22—W1—P1—C3	3.0 (2)	Cl1—W1—P3—C9	−98.7 (2)
Cl21—W1—P1—C3	179.16 (18)	P1—W1—P3—C9	81.82 (19)
Cl1—W1—P1—C3	−92.92 (18)	P2—W1—P3—C9	88.4 (2)
P3—W1—P1—C3	86.56 (18)	P4—W1—P3—C9	−91.47 (19)
P2—W1—P1—C3	−89.93 (18)	N11—W1—P3—C7	−68.8 (3)
P4—W1—P1—C3	99.15 (18)	N12—W1—P3—C7	115.6 (5)
N11—W1—P1—C4	−129.7 (3)	Cl22—W1—P3—C7	−69.9 (2)
N12—W1—P1—C4	48.6 (5)	Cl21—W1—P3—C7	113.84 (19)
Cl22—W1—P1—C4	−128.9 (3)	Cl1—W1—P3—C7	16.37 (19)
Cl21—W1—P1—C4	47.2 (2)	P1—W1—P3—C7	−163.11 (18)
Cl1—W1—P1—C4	135.1 (2)	P2—W1—P3—C7	−156.49 (18)
P3—W1—P1—C4	−45.4 (2)	P4—W1—P3—C7	23.60 (18)
P2—W1—P1—C4	138.1 (2)	N11—W1—P4—C12	−154.6 (3)
P4—W1—P1—C4	−32.8 (2)	N12—W1—P4—C12	29.4 (5)
N11—W1—P1—C1	117.5 (3)	Cl22—W1—P4—C12	−155.9 (2)
N12—W1—P1—C1	−64.3 (4)	Cl21—W1—P4—C12	29.8 (2)
Cl22—W1—P1—C1	118.2 (2)	Cl1—W1—P4—C12	−64.1 (2)
Cl21—W1—P1—C1	−65.69 (18)	P3—W1—P4—C12	120.7 (2)
Cl1—W1—P1—C1	22.23 (19)	P1—W1—P4—C12	107.9 (2)
P3—W1—P1—C1	−158.29 (17)	P2—W1—P4—C12	−59.2 (2)
P2—W1—P1—C1	25.23 (17)	N11—W1—P4—C11	−29.1 (3)
P4—W1—P1—C1	−145.69 (18)	N12—W1—P4—C11	154.9 (5)
N11—W1—P2—C5	30.3 (3)	Cl22—W1—P4—C11	−30.3 (2)
N12—W1—P2—C5	−154.9 (5)	Cl21—W1—P4—C11	155.4 (2)
Cl22—W1—P2—C5	31.7 (2)	Cl1—W1—P4—C11	61.5 (2)
Cl21—W1—P2—C5	−153.9 (2)	P3—W1—P4—C11	−113.7 (2)
Cl1—W1—P2—C5	−59.4 (2)	P1—W1—P4—C11	−126.5 (2)
P3—W1—P2—C5	115.8 (2)	P2—W1—P4—C11	66.4 (2)
P1—W1—P2—C5	122.6 (2)	N11—W1—P4—C8	87.0 (3)
P4—W1—P2—C5	−64.3 (2)	N12—W1—P4—C8	−89.0 (4)
N11—W1—P2—C6	153.4 (3)	Cl22—W1—P4—C8	85.7 (2)
N12—W1—P2—C6	−31.9 (5)	Cl21—W1—P4—C8	−88.5 (2)
Cl22—W1—P2—C6	154.8 (2)	Cl1—W1—P4—C8	177.56 (19)
Cl21—W1—P2—C6	−30.9 (2)	P3—W1—P4—C8	2.37 (19)
Cl1—W1—P2—C6	63.6 (2)	P1—W1—P4—C8	−10.4 (2)
P3—W1—P2—C6	−121.1 (2)	P2—W1—P4—C8	−177.56 (19)
P1—W1—P2—C6	−114.4 (2)	C3—P1—C1—C2	68.8 (4)
P4—W1—P2—C6	58.8 (2)	C4—P1—C1—C2	177.6 (4)
N11—W1—P2—C2	−91.2 (3)	W1—P1—C1—C2	−54.9 (4)
N12—W1—P2—C2	83.6 (5)	P1—C1—C2—P2	53.9 (4)
Cl22—W1—P2—C2	−89.8 (2)	C5—P2—C2—C1	−160.7 (3)
Cl21—W1—P2—C2	84.53 (19)	C6—P2—C2—C1	89.0 (4)
Cl1—W1—P2—C2	179.05 (18)	W1—P2—C2—C1	−32.2 (4)
P3—W1—P2—C2	−5.7 (2)	C10—P3—C7—C8	−179.3 (3)
P1—W1—P2—C2	1.03 (18)	C9—P3—C7—C8	71.3 (4)
P4—W1—P2—C2	174.18 (18)	W1—P3—C7—C8	−53.8 (3)
N11—W1—P3—C10	43.7 (3)	P3—C7—C8—P4	54.2 (4)
N12—W1—P3—C10	−132.0 (5)	C12—P4—C8—C7	−158.3 (3)
Cl22—W1—P3—C10	42.6 (2)	C11—P4—C8—C7	89.3 (4)
Cl21—W1—P3—C10	−133.7 (2)	W1—P4—C8—C7	−33.7 (4)
Cl1—W1—P3—C10	128.8 (2)	Cl22—W1—N11—O11	2(10)
P1—W1—P3—C10	−50.6 (2)	Cl1—W1—N11—O11	174 (4)
P2—W1—P3—C10	−44.0 (2)	P3—W1—N11—O11	−44 (4)
P4—W1—P3—C10	136.1 (2)	P1—W1—N11—O11	32 (4)
N11—W1—P3—C9	176.1 (3)	P2—W1—N11—O11	105 (4)
N12—W1—P3—C9	0.5 (5)	P4—W1—N11—O11	−118 (4)
